# Does Radar Technology Support the Diagnosis of Pneumothorax? PneumoScan—A Diagnostic Point-of-Care Tool

**DOI:** 10.1155/2013/489056

**Published:** 2013-09-25

**Authors:** T. Lindner, M. Conze, C. E. Albers, B. A. Leidel, P. Levy, C. Kleber, M. De Moya, A. Exadaktylos, C. Stoupis

**Affiliations:** ^1^Department of Emergency Medicine, Charité-Universitätsmedizin, Berlin, Germany; ^2^Department of Emergency Medicine, University Hospital-Inselspital, Bern, Switzerland; ^3^Department of Emergency Medicine, Wayne State University, Detroit, MI, USA; ^4^Center for Musculoskeletal Surgery, Charité-Universitätsmedizin, Berlin, Germany; ^5^Division of Trauma, Emergency Surgery & Surgical Critical Care, Massachusetts General Hospital, Boston, MI, USA; ^6^Department of Radiology, Spital Männedorf, Männerdorf, Switzerland

## Abstract

*Background*. A nonrecognized pneumothorax (PTX) may become a life-threatening tension PTX. A reliable point-of-care diagnostic tool could help in reduce this risk. For this purpose, we investigated the feasibility of the use of the PneumoScan, an innovative device based on micropower impulse radar (MIR). *Patients and Methods*. addition to a standard diagnostic protocol including clinical examination, chest X-ray (CXR), and computed tomography (CT), 24 consecutive patients with chest trauma underwent PneumoScan testing in the shock trauma room to exclude a PTX. *Results*. The application of the PneumoScan was simple, quick, and reliable without functional disorder. Clinical examination and CXR each revealed one and PneumoScan three out of altogether four PTXs (sensitivity 75%, specificity 100%, positive predictive value 100%, and negative predictive value 95%). The undetected PTX did not require intervention. *Conclusion*. The PneumoScan as a point-of-care device offers additional diagnostic value in patient management following chest trauma. Further studies with more patients have to be performed to evaluate the diagnostic accuracy of the device.

## 1. Introduction

Thoracic trauma is frequent in multiple trauamatized patients. According to the current annual report of the TraumaRegister of the German Trauma Society (DGU) 56% of 10766 documented severe trauma patients with *Injury Severity Score* (ISS) ≥ 16 points showed thoracic injuries with an *Abbreviated Injury Scale* (AIS) ≥ 3 points [1]. Beside rib fractures, lung contusion and PTX are the main consequences of blunt chest trauma. In hospital, significant PTX is detected between 37 and 59% of the cases [2]. Primary routine diagnostics in shock trauma room include a clinical examination and conventional CXR. However, a significant percentage of PTX maintains undetected by these methods and is first distinguished by CT scan. The number of occult PTXs range from 2 to 15% [3–5], in some studies even 50% [6]. Therefore the “S-3 guideline on treatment of patients with severe and multiple injuries of the DGU” recommends expanding radiologic diagnostics by thoracic ultrasound (eFAST) when suspecting thoracic trauma. If significant clinical signs are present a thoracic CT scan with i.v. contrast agent is advised, alternatively even primary [2]. CXR, and CT scan only are available in hospital and ultrasound is not used regularly in the prehospital setting. Therefore, only mechanism of injury, and clinical examination with assessment of ventilation can be consulted for diagnosing or excluding PTX. Due to low sensitivity (43–90%) and specificity (79–98%) of each single criterion, only their combination allows secure assessment [2, 7, 8].

Circumstances as mass casualty incident (MCI) could rapidly restrict clinical established diagnostics by limiting available resources. Further on, a CBRN (chemical, biological, radiological, and nuclear) attack can cause massive delay of treatment, as decontamination of victims is the primary focus.

The initial, secure, and fast detection or exclusion of PTX has however a high impact on the management, especially if continuous monitoring and immediate treatment of PTX through chest tube placement cannot be guaranteed [2, 7, 8].

An anytime bedside available, examiner-independent, easy to use, and fast method with high diagnostic accuracy in terms of a point-of-care device to rule out PTX would be desirable [7]. Therefore, an innovative and nonionizing micropower impulse radar- (MIR-) based tool—the PneumoScan engineered by PneumoSonic Inc. (Cleveland, OH, USA)—is investigated to exclude PTX in the context of shock trauma room treatment of severely injured patients.

## 2. Patients and Methods

### 2.1. PneumoScan

Based on micropower impulse radar (MIR), the PneumoScan is a portable, battery-powered, CE-certified (CE certificate 561036) diagnostic tool, available via PneumoSonic Inc. (Cleveland, OH, USA). It emits extremely low-power ultrashort electromagnetic signals with a frequency of 500 megahertz to six gigahertz [9]. Those ultrawideband (UWB) signals can penetrate different materials like human tissue. Each tissue (e.g., fat, muscle, and bone) reflects these waves differently. The in-built receiver of PneumoScan analyses those specific reflections. Thereby, abnormal conditions like a PTX can be detected [10]. The Lawrence Livermore National Laboratory (Livermore, CA, USA) examined the physiologic reflection pattern of the lung in healthy patients and compared the results to those with PTX condition. Thereby, baseline data were gained and defined. By using specific algorithms the software of PneumoScan correlates received patient-specific signals with baseline data [10]. 

Spatial accuracy of PneumoScan amounts approximately five millimetres, penetration depth approximately seven centimetres. The impulses also can penetrate textiles to allow an examination of a dressed patient [11]. Transients of other technical devices are suppressed by combination of broad frequencies, low power demand, and emission of very short impulses. The functions of electronic devices such as a cardiac pacemaker are not affected.

**Figure 1:**
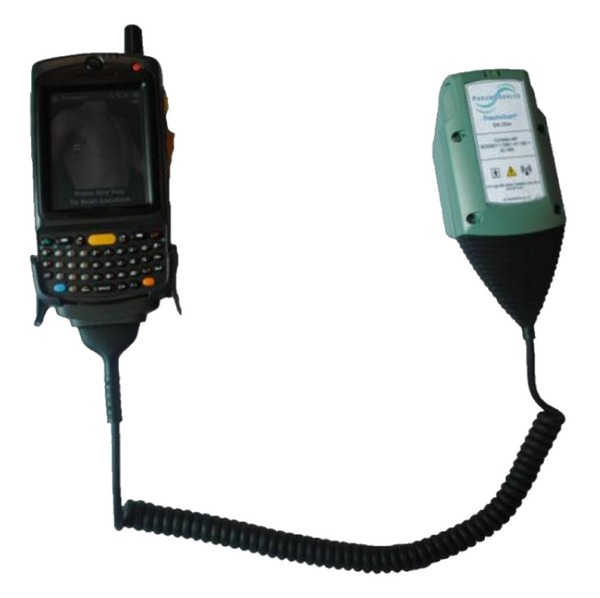
PneumoScan device.

The device consists of two pieces: the MIR transceiver with antenna and processor (Figure 1, right), as well as a portable handheld computer, for example, Motorola MC75 (Figure 1, left).

**Figure 2:**
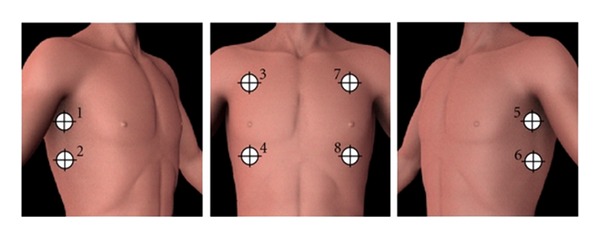
PneumoScan data acquisition points.

**Figure 3:**
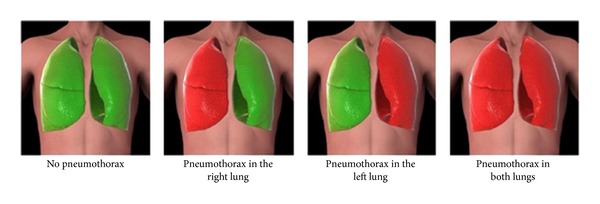
Display of results: red = PTX, green = no PTX.

The PneumoScan analyses signals of eight specific sites along the anterior thorax (Figure 2). Total scan time is one to two minutes. Each correct scan is signalized visually and acoustically. After eight completed scans, the result with localisation (right or left lung) is displayed by red (PTX) or green (no PTX) colour coding (Figure 3).

### 2.2. Study Procedure

Between May and June 2011, 24 severely injured adult patients with blunt or penetrating chest trauma, admitted via shock trauma room, were included in the survey in a level-one trauma centre. Treatment was standardized according to *Advanced Trauma Life Support* (ATLS) protocol. Primary imaging diagnostics were conducted in shock trauma room by CXR (Lodox Statscan, Lodox Systems (Pty) Ltd, Benmore, South Africa) and secondarily after shock trauma treatment by full body spiral CT with contrast agent (Somatom Sensation 16, Siemens AG, Erlangen, Germany) [12].

Referring to our question, application of PneumoScan to exclude a PTX was conducted additionally in shock trauma room. Scans were performed by two physicians and two medical students after a 15-minute instruction tutorial. PneumoScan measurements took place during clinical examination, but before CXR and CT scan (scans on all included patients were performed within first 15 minutes), to avoid falsification of results through a potentially growing PTX in following examinations. PneumoScan results were blinded to the examiner (no real-time display of the results) and performed without knowledge of CXR and CT findings.

Chest CT was used as gold standard in all examinations. By CT scan detected PTX were classified through their maximal extension in axial slices (maximal distance of visceral pleura to parietal pleura) and through their position (anterior/lateral/posterior).

## 3. Results

In total, 24 severely injured patients (ISS ≥ 3 points) with nearly exclusive blunt chest trauma (96%) were enrolled. Eighty-eight percent were male. Mean age was 47 years (Table 1).

Four one-sided PTXs were diagnosed by CT scan, three of them on the right side, one on the left side. Clinical examination and CXR detected one PTXs. Through PneumoScan, three PTXs were detected, whereof two PTXs were clinically significant (treated with chest tube placement) (Table 2).

One maximal 20-millimetre large anterior PTX with no need for chest tube placement was neither detected by PneumoScan nor by clinical examination or CXR.

The sensitivity of PneumoScan measurements amounted to 75%, with specificity 100%. The prevalence of PTX was 17%. The positive predictive value was 100% and negative predictive value 95% (Table 3).

## 4. Discussion

In our study we were able to show that the MIR-based PneumoScan as a point-of-care device could be a helpful tool for detecting PTX, in context of shock trauma room management following ATLS protocol. Despite low number of cases, we revealed at least a principle advantage of PneumoScan towards clinical examination and CXR in excluding PTX, which could increase diagnostic safety. The results of this study with 24 applied patients underline the good findings of a previous study with 50 patients to assess the diagnostic value of PneumoScan (sensitivity 85.7% (1/7 false negative), specificity 97.7% (1/43 false positive), gold standard CT [13]).

The only undetected PTX by PneumoScan was neither revealed by clinical examination nor by CXR. The body mass-index (BMI) of this patient (lowest BMI of all enrolled patients) contradicts considerations that PneumoScan might not penetrate tissue deep enough (penetration depth of PneumoScan: seven centimetres). Refering to CT, maximum extension of the unrevealed PTX was larger than the smallest PTX detected by the device (spatial accuracy of PneumoScan: five millimetres). One could assume that this particular PTX developed itself in time course. The undetected PTX was not significant (no chest tube placement needed) in clinical course.

On scene, emergency physicians and emergency medical technicians (EMT) can only adequately detect a traumatic PTX by combining single findings of clinical examination [7, 8]. Even if present studies report that a wait-and-see attitude in occult PTX (even in ventilated patients) can be as safe as chest tube placement [14, 15], it is not clear which kind of cases are applicable to this statement [5]. In principle, every PTX can develop life-threatening complications any time. An early and safe exclusion or detection of PTX would constitute an advantage in treatment, in terms of a better risk assessment of the patient. Focussed assessment sonography for trauma (FAST) is integrated in ATLS protocol to exclude free abdominal fluid. By simply expending the examination to the chest, ultrasound could be used for PTX diagnostics, also in the preclinical setting. However, different studies showed high specificity (approximately 99%), but fluctuating sensitivity (47–100%, pooled sensitivity: 88%) [16]. Furthermore, ultrasound diagnostic is highly examiner-dependent and not practicable for untrained personnel [16–19]. Especially in case of mass casualties rescue teams need to triage patients in a very limited time. Therefore, a reliable and compact point-of-care diagnostic tool with an easy handling is desirable [7]. In a previous study examining the diagnostic value of PneumoScan, all measurements were conducted by only two physicians [13]. In the present study two medical students were able to perform and interpret readings with the PneumoScan only after a 15-minute tutorial which definitely states an advantage towards ultrasound. Regarding the frequency of traumatic PTX [2] and the diagnostic weakness of CXR [3–6], PneumoScan even seems to be a reasonable addition or alternative to chest ultrasound in the primary survey (Breathing) of shock trauma room management. Improved preclinical diagnostic possibilities may reduce “preventive” chest tube placements which might cause unnecessary delay and complications [20, 21]. Nonmedical staff could complement the field of operation, whereby PTX could be detected much earlier and development of life-threatening tension PTX could be avoided.

## 5. Conclusion

Further clinical and preclinical surveys with a bigger population of patients are required to evaluate the diagnostic accuracy of PneumoScan in detection of PTX. Basically, the MIR-powered device offers a fast point-of-care method, which on top is easy to use only after a short tutorial. Beside shock trauma room management, especially preclinical use and disaster medicine are potential fields of operation.

## Figures and Tables

**Table 1:** Patient characteristics.

Patients total (%)	24 (100)
Male patients (%)	21/24 (88)
Mean age; min–max (years)	47; 18–87
Blunt chest trauma ratio	23/24 (96)

min:  minimum, max:  maximum.

**Table 2:** Summary of results and patient characteristics with CT scan affirmed PTX.

Pat	Age	Sex	BMI (kg/m²)	Trauma	Ribfx	Side of PTX (in CT)	Location of PTX (in CT)	Size of PTX (in mm)	Clinical exam	CXR Lodox	Pneumo-Scan	Chest tube
1	72	m	29	Blunt	Right	Right	Ant	10	+	Ø	+	+
3	33	m	22	Blunt	Right	Right	Ant	20	Ø	Ø	Ø	Ø
8	49	m	27	Blunt	Right	Right	Ant and post	24	Ø	Ø	+	Ø
9	43	m	23	Blunt	No	Left	Ant	47	Ø	+	+	+

+: yes/positive; Ø: no/negative; m: male; PTX:  pneumothorax; CT:  computer tomography; ant:  anterior; post:  posterior; size of PTX: major distance between visceral-  and parietal pleura in axial CTscan.

**Table 3:** Diagnostic value of investigated diagnostics; all data in percent.

	Sensitivity	Specificity	PPV	NPV
Clinical exam	25	100	100	88
CXR	25	100	100	88
PneumoScan	75	100	100	95
CT	100	100	100	100

PPV: positive predictive value; NPV: negative predictive value; prevalence: 17%.

## References

[1] Lefering R, Paffrath T, Nienaber U TraumaRegister DGU—Jahresbericht. http://www.traumaregister.de.

[2] http://www.awmf.org/leitlinien.

[3] Ball CG, Kirkpatrick AW, Feliciano DV (2009). The occult pneumothorax: what have we learned?. *Canadian Journal of Surgery*.

[4] Ball CG, Kirkpatrick AW, Laupland KB (2005). Incidence, risk factors, and outcomes for occult pneumothoraces in victims of major trauma. *Journal of Trauma*.

[5] Moore FO, Goslar PW, Coimbra R (2011). Blunt traumatic occult pneumothorax: is observation safe?—results of a prospective, AAST multicenter study. *Journal of Trauma*.

[6] Soldati G, Testa A, Sher S, Pignataro G, La Sala M, Silveri NG (2008). Occult traumatic pneumothorax: diagnostic accuracy of lung ultrasonography in the emergency department. *Chest*.

[7] Waydhas C (2012). Preclinical management of multiples injuries: S3 guideline. *Unfallchirurg*.

[8] Waydhas C, Sauerland S (2007). Pre-hospital pleural decompression and chest tube placement after blunt trauma: a systematic review. *Resuscitation*.

[9] Azevedo S, McEwan TE (1997). Micropower impulse radar: a new pocket-sized radar that operates up to several years on AA batteries. *IEEE Potentials*.

[10] Meissner C (2009). The next generation of medical diagnostic devices. *Science and Technology Review*.

[11] Levy PD, Wielinski T, Greszler A (2011). Micropower impulse radar: a novel technology for rapid, real-time detection of pneumothorax. *Emergency Medicine International*.

[12] Exadaktylos AK, Benneker LM, Jeger V (2008). Total-body digital X-ray in trauma. An experience report on the first operational full body scanner in Europe and its possible role in ATLS. *Injury*.

[13] Albers CE, Haefeli PC, Zimmermann H, de Moya M, Exadaktylos AK (2013). Can handheld micropower impulse radar technology be used to detect pneumothorax? Initial experience in a European trauma centre. *Injury*.

[14] Yadav K, Jalili M, Zehtabchi S (2010). Management of traumatic occult pneumothorax. *Resuscitation*.

[15] Mowery NT, Gunter OL, Collier BR (2011). Practice management guidelines for management of hemothorax and occult pneumothorax. *Journal of Trauma*.

[16] Ding W, Shen Y, Yang J, He X, Zhang M (2011). Diagnosis of pneumothorax by radiography and ultrasonography: a meta-analysis. *Chest*.

[17] Morgan AR, Vasios WN, Hubler DA, Benson PJ (2010). Special operator level clinical ultrasound: an experience in application and training. *Journal of Special Operations Medicine*.

[18] Soldati G, Giunta V, Sher S, Melosi F, Dini C (2011). ‘Synthetic’ comets: a new look at lung sonography. *Ultrasound in Medicine and Biology*.

[19] Soldati G, Sher S, Testa A (2011). Lung and ultrasound: time to ‘reflect’. *European Review for Medical and Pharmacological Sciences*.

[20] Aufmkolk M, Ruchholtz S, Hering M, Waydhas C, Nast-Kolb D (2003). Wertigkeit der subjektiven Einschätzung der Thoraxverletzungsschwere durch den Notarzt. *Der Unfallchirurg*.

[21] Aylwin CJ, Brohi K, Davies GD, Walsh MS (2008). Pre-hospital and in-hospital thoracostomy: indications and complications. *Annals of the Royal College of Surgeons of England*.

